# Clinical Course of Development of Alcohol and Opioid Dependence: What are the Implications in Prevention?

**DOI:** 10.4103/0970-0218.66895

**Published:** 2010-04

**Authors:** Sahoo Saddichha, Narayana Manjunatha, Christoday Raja Jayant Khess

**Affiliations:** National Institute of Mental Health and Neurosciences (NIMHANS), Bangalore, India; 1 Central Institute of Psychiatry, Ranchi, India

## Introduction

In India, it is estimated that 75 million people are alcohol users and nearly 3 million are opioid users.(1) Of these, there has been a noted prevalence of 19.78-21.4%(2) of alcohol use and 5% of alcohol dependence in Indian population.(3) The prevalence of opium use in India has also been increasing and it is now considered to be a ’party drug’ or ’relaxation drug’. Several studies have described the prevalence of opium abuse to be 1.51-2%(3,4) although a recent study notes it to be around 0.4%.(2)

Yet, there is a concerning increase in the social acceptance of alcohol even for frequent self-induced intoxication and easier access to ’hard drugs’ like opioids is now responsible for driving adolescents toward substance use and a trend is being noted toward lower ages of onset of both alcohol and opioid use. Even though opium use is generally frowned upon, alcohol use is widely accepted. There is, therefore, an urgent need for reduction in the demand of drugs of addiction, both legal and illegal, which may otherwise lead to numerous health, family and societal consequences.

One of the ways this can be made possible is by identifying and preventing the development of dependence in both alcohol and opioid users. This study is therefore aimed at:

- Studying the clinical course of development, in terms of ages, order of onset and duration of criteria of ICD-10 dependence, of both alcohol and opioid dependence.
- Comparing and contrasting the two substances to evaluate differences if any, to formulate a strategy for primary prevention.

## Materials and Methods

Consecutively admitted patients of ≥18 years of age for treatment of dependence in the period of August 2005 to May 2006 in Centre for Addiction Psychiatry, Central Institute of Psychiatry, Ranchi, India with ICD-10 DCR(5) diagnosis of alcohol dependence syndrome or opioid dependence syndrome (made by a senior resident/senior consultant) and giving written informed consent were recruited for the study. Subjects with other co-morbid psychiatric disorders/substance dependence/general medical condition and with MMSE score <24 and were excluded from the study.

All the subjects participated in the personal face to face interview after medically supervised withdrawal using alcohol or other drug (opioid) section of SSAGA-II(6) (revised in 1997) according to individual diagnosis of patient. The details of this instrument and the methods have been given in an earlier paper.(7)

Since it was a retrospective recall study, questions were framed individually to trigger the recall with reasonable accuracy using anchor questions to memorable events, tagging the questions with specific examples and defining the technical terms.(8) All ratings were done by an investigator blind to the diagnosis and current status of the subjects. The data were statistically analyzed by means of T-test for descriptive variables and the Chi-squared test for categorical variables.

## Results

Total sample size of the present study was 112 of which 81(72%) were alcohol dependents and 31 (28%) were opioid dependents. All patients were males.

Table 1 summarizes the socio-demographic characteristics compared across the two groups. Statistical differences were found in age of the patients and marital status between both groups but not in residence, education and occupation. Table 2 presents the clinical course of development of dependence between the two groups. Mean age at onset of alcohol use in this study was 18.72 (+ 6.84) years as compared to opioid use being initiated at 20.73 (± 3.93) years which was statistically significant (*P*=0.05). The other significant findings are detailed in Table 2.

**Table 1:** Socio-demographic characteristics of study samples

Characteristics	Alcohol users group (*N*=81)	%	Opioid users group (*N*=31)	%	Chi square/T-test	Df	Significance
Age	35.16 ± 10.2		26.09 ± 5.65		4.670	110	<0.001**
Residence						
Rural	20	24.7	3	9.7	1.442	110	0.152
Urban	61	75.3	28	90.3			
Education	11.69 ± 3.98		10.51 ± 3.49		3.230	1	0.072
Occupation						
Professional and semi-professional	22	27.2	3	9.7	8.293	4	0.081
Skilled and semi-skilled	40	49.4	20	64.5			
Unemployed	7	8.6	2	6.5			
Retired	5	6.2	0	0			
Students	7	8.6	6	19.4			
Marital Status						
Single	22	27.2	19	61.3	11.547	3	0.009*
Married	57	70.4	12	38.7			
Widowed	1	1.2	0	0			
Separated	1	1.2	0	0			

T-test has been used for continuous variables and Chi-square for categorical variables.

* Significance at *P*<0.05

** Significance at *P*<0.001

**Table 2:** Clinical course of development of dependence

Factors	Alcohol-numbers (%)/ mean ± S.D	Opioid-numbers (%)/mean ± S.D	Chi square/t-value	df	Significance
Age of onset of substance use	18.72 ± 6.84	20.73 ± 3.93	1.937	110	0.05*
Age of appearance of first criteria	24.33 ± 9.21	21.39 ± 3.94	1.710	110	0.09
Age of appearance of ICD 10 dependence	27.51 ± 9.28	22.05 ± 3.98	3.159	110	0.002*
Duration from onset to development of first criteria	5.61 ± 6.2	0.74 ± 0.13	4.147	110	<0.001**
Duration from first criteria to dependence	3.17 ± 3.23	0.65 ± 0.56	4.288	110	<0.001**
Duration from onset to dependence	8.78 ± 6.7	1.32 ± 0.89	6.162	110	<0.001**
Use in first degree relatives						
Dependence	63 (77.8)	2 (6.5)	46.833	1	<0.001**
No substance use	18 (22.2)	29 (93.5)			
Most common first criteria						
Tolerance	58 (71.6)	13 (41.9)	32.155	4	<0.001**
Withdrawal	6 (7.4)	17 (54.8)			
Loss of control	3 (3.7)	0			
Persistent use despite knowledge	1 (1.2)	0			
Craving	13 (11.6)	1 (3.2)			

T-test has been used for continuous variables and Chi-square for categorical variables.

* Significance at *P*<0.05

** Significance at *P*<0.001

Figures in parenthesis are percentages

The most common first criteria to appear in alcohol users was tolerance (71.6%) followed by second criteria as craving (11.6%) whereas, in the opioid group, withdrawal symptoms (54.8%) were the most common followed by tolerance (41.9%), which was statistically significant (*P*<0.001).

## Discussion

India, as a rapidly growing economy, is also being besieged by problems more familiar with Western audiences, such as rampant alcohol and drug dependence. Along with the steady rise in per capita income, there has been an associated rise in both alcohol and opioid use. The present study revealed that the mean duration (in years± SD) between onset of alcohol use and development of first criterion is 5.61 (± 6.2) years providing a ’window of opportunity’ for prevention toward further development of dependence. This becomes more imperative in the opioid group where the mean duration is only 0.74 (± 0.13) years. The mean duration in both groups also reflects the period of ’criteria-free or social use’ which is longer in alcohol users than opioid users, thereby limiting the time duration during which such users can be targeted for intervention.

One of the important findings of this study has been the fact that, although opioid use has a very rapid and expected transition from onset to dependence, a rapid but unexpected transition takes place in alcohol use also, which has been observed in earlier studies.(9) However, alcohol misuse is not legally prohibited,(10) in spite of the fact that it has massive social, economic and medical consequences and is responsible for a large proportion of the healthcare burden in almost all populations.(11) Similarly, the transition from opioid use to dependence carries a dire prognosis with a 2% risk of dying every year and a high mortality rate of about 50% in a 30 year follow-up and also corroborated by a 20 year follow-up study.(12) The short duration from onset to dependence in opioids and common experience of tolerance and withdrawal symptoms seen with our study along with poor outcome is a clarion call to renew efforts to strengthen regulative measures. In fact, most opioids reported for illicit use are prescription opioids and thus originate directly or indirectly from the medical system or are diverted from therapeutic use.(13,14) Perhaps, the solution lies in reforming drug use laws and focusing on monitoring and regulating medical opioids with a goal to eradicating illicit use.(15)

A comparison with other studies from around the globe reveals the usual age of onset of alcohol use to be around 15 years (18 years in our study) with earlier onset associated with higher dependence rates.(11) Heavy drinking is usually between 18 and 22 years of age (24 years in our study) with dependence occurring in the early to mid-20s(11,16) (27 years in our study). Similarly opioid abuse may begin as early as 12 years (20 years in our study), with dependence being more common among males in the early thirties(17) (22 years in our study). This reflects a later age of onset and dependence among alcohol users in this country but a more rapid development of dependence among opioid users when compared to other studies.

Although these findings were noted only among males (our sample being entirely male), we believe that drug use may not be limited to a single gender. However, other studies from different centers in this country have observed that females with drug use rarely come for treatment or de-addiction,(18) contrary to observations made in North America and other countries.(19) This phenomenon exists despite widespread drug use noted in the community,(9) due to which we also had similar problems of enrolling female subjects. Owing to poor participation, we eventually had to drop them from the study and include only male subjects for our final analysis.

## Conclusion

Alcohol and opioid use follow a somewhat different clinical course in the development of dependence syndrome. Therefore, a ’one size fits all’ approach may not work. Further, primary prevention is indeed feasible for both alcohol and opioid dependence by GPs and health workers routinely enquiring about substance use. Early specialist referrals are preferable, especially in opioid use, even before the development of clinical dependence syndrome (to manage withdrawal symptoms by specialists).
